# Cost‐effectiveness of hydrophilic‐coated urinary catheters for individuals with spinal cord injury: A systematic review

**DOI:** 10.1002/bco2.63

**Published:** 2020-12-20

**Authors:** Min Xi, Dean S. Elterman, Blayne Welk, Maureen Pakosh, Brian C. F. Chan

**Affiliations:** ^1^ KITE ‐ Toronto Rehabilitation Institute University Health Network Toronto ON Canada; ^2^ Institute of Health Policy, Management, and Evaluation University of Toronto ON Canada; ^3^ Division of Urology Department of Surgery University Health Network Toronto ON Canada; ^4^ Department of Surgery Western University London ON Canada; ^5^ Library & Information Services University Health Network Toronto Rehabilitation Institute Toronto ON Canada

## Abstract

**Objective:**

To identify and critically evaluate the economic evaluations examining the cost‐effectiveness of hydrophilic‐coated vs uncoated catheters for individuals with spinal cord injury.

**Methods:**

We searched MEDLINE, the Excerpta Medica database (EMBASE), Cochrane Database of Systematic Reviews, the Cumulative Index to Nursing and Allied Health Literature (CINAHL), and Emcare for studies in English and French. There were no restrictions to the year of publication. Our search strategy included the following key terms: “spinal cord injury,” “catheterization,” and “cost analysis.”

**Results:**

The search identified 371 studies, of which eight studies met the inclusion criteria. Five studies observed hydrophilic‐coated catheters to be cost‐effective compared to uncoated catheters. Two studies found hydrophilic‐coated catheters to be not cost‐effective compared to uncoated catheters and one study estimated that hydrophilic‐coated catheters reduced the long‐term health‐care costs compared to uncoated catheters.

**Conclusion:**

The cost‐effectiveness of hydrophilic‐coated catheters was dependent on the comparator used, the consideration of long‐term effects, and the unit cost of treatment. Further studies are needed to explore the short‐term and long‐term effects of hydrophilic‐coated catheter use on urinary tract infections and clarify the impact of hydrophilic‐coated catheter use on long‐term renal function. Overall, our critical evaluation of the literature suggests that the evidence is pointing toward hydrophilic‐coated catheters being cost‐effective, particularly when a societal perspective is applied.

## 1 INTRODUCTION

A large proportion of individuals with spinal cord injuries suffer from neurogenic bladder. In the United States, neurogenic bladder affects 80% of the 12 000 patients with new spinal cord injuries per year.^1^, ^2^ Of these individuals, more than 80% will need some form of urinary catheterization.^3^

Individuals requiring catheterization have several options broadly falling into two categories: indwelling and intermittent catheters. Indwelling catheters are inserted via the urethra or the abdomen (supra‐pubic) and are left in situ. Urine is collected through an attached drainage bag.^4^ After insertion, indwelling catheters can remain in the bladder for an extended period of time and are regularly changed every 4‐6 weeks.^5^ However, indwelling catheters have been shown to have a higher number of complications, including bladder stones, urinary tract infection (UTI), and decreased bladder capacity, in comparison to intermittent catheters.^6^, ^7^, ^8^ Intermittent catheterization is recommended as the gold standard for bladder management for individuals with spinal cord injury and offers several advantages over indwelling catheters.^9^, ^10^ Intermittent catheters are inserted via the urethra or the abdomen, similar to indwelling catheters.^4^ In contrast to indwelling catheters, intermittent catheters can be inserted by the patient or caregiver in any location and are immediately removed after bladder drainage.^4^ Other advantages of intermittent catheters include lower risk of UTI and other complications, increased quality of life and patient autonomy, and fewer social and intimacy barriers.^9^, ^10^ Additionally, intermittent catheters have higher rates of patient adherence. A study by Cameron et al found that only 71% of individuals continue to use intermittent catheters 30 years after initial use.^11^

Among the different types of intermittent catheters, uncoated and single‐use hydrophilic‐coated catheters are the most commonly used. Uncoated catheters are often made from medical‐grade polyvinyl chloride (PVC) and require manual external gel lubrication prior to insertion.^12^ The need to manually self‐lubricate may be particularly difficult for individuals with limited upper limb function.^13^ Catheterization with uncoated catheters may have a slightly increased risk of bacterial infection and other complications such as hematuria.^14^, ^15^ Moreover, hydrophilic‐coated catheters are coated with polyvinylpyrrolidone, a polymer that creates a lubricated surface upon exposure to water to facilitate the insertion of the catheter. Therefore, hydrophilic‐coated catheters do not require manual lubrication prior to insertion.^16^ Moreover, hydrophilic‐coated catheters may lead to better health outcomes due to reduced risk of infection. A previous systematic review and meta‐analysis identified a 16% reduction in UTI risk associated with hydrophilic‐coated catheter use in comparison to uncoated catheter use.^17^ However, the clinical benefits of hydrophilic‐coated catheters come at a cost since this the unit cost for this technology is greater than that of uncoated catheters.^4^, ^18^

Several studies have examined the cost‐effectiveness of hydrophilic‐coated vs uncoated catheters. Two reviews on this topic have been conducted. One review was conducted by Health Quality Ontario (HQO) in 2019.^4^ This review sought to determine the cost‐effectiveness of intermittent catheterization for long‐term usage and identified papers published prior to 2016.^4^ All five identified studies comparing hydrophilic‐coated and uncoated catheters found hydrophilic‐coated catheters to be a cost‐effective option.^4^ Another review was conducted by Saadat et al and identified six papers from 2014 to 2018; they reviewed the cost‐effectiveness of single‐use vs repeated‐use catheters and hydrophilic‐coated vs uncoated catheters. This study briefly summarized the results of each included paper but did not make any conclusions on cost the effectiveness of hydrophilic‐coated catheters.^19^ Neither of these two previous studies critically evaluated the economic evaluations included in the review.

In order to fill the knowledge gaps identified in the existing literature, our systematic review sought to identify and critically evaluate the economic evaluations examining the cost‐effectiveness of hydrophilic‐coated vs uncoated catheters for individuals with spinal cord injury. The findings of our study can be used to provide information for public health‐care payers to determine whether hydrophilic‐coated catheters should be publicly funded.

## 2 METHODS

### 2.1 Search strategy and studies identification

We searched MEDLINE, the Excerpta Medica database (EMBASE), Cochrane Database of Systematic Reviews, the Cumulative Index to Nursing and Allied Health Literature (CINAHL), and Emcare. There were no restrictions to the year or to the language of the publication. Our search strategy included the following key terms: “spinal cord injury,” “catheterization,” and “cost analysis.” The full search strategy is included in Appendix A. We also conducted a bibliographic hand search of all review articles identified in the database search.

Publications were included if they: (1) included a full economic evaluation (eg, cost‐effectiveness, cost‐utility, or cost‐benefit study) of any type of urinary catheter; (2) were conducted for individuals with spinal cord injury; and (3) were written in English or French. We excluded gray literature, conference abstracts, systematic reviews, comprehensive reviews, letters, guidelines, news articles, and policy analyses. We also excluded costing studies and cost comparison analyses.

After the removal of duplicates, titles and abstracts were independently screened by BC and MX using Covidence software.^20^ Potentially relevant full‐text articles were screened by BC and MX for an inclusion or exclusion decision. Discrepancies during abstract and full‐text screening were resolved by BC.

### 2.2 Data collection and analysis

We evaluated the methodological quality of the included articles using the Drummond checklist.^21^ The Drummond checklist has been recommended for assessing the quality of economic evaluation studies and includes four main categories: study design, data collection, analysis, and interpretation of results. We used the Drummond checklist for quality assessment due to its broad applicability to various types of economic evaluation studies and its simple structure.^22^, ^23^ The quality assessment was carried out by two reviewers (BC and MX) and discrepancies were resolved through consensus between the two reviewers.

BC and MX extracted data from the included articles. Extracted variables included study characteristics (eg, author, country study design, year of publication, population, time horizon, intervention, and comparator) and outcomes of interest (eg, costs and incremental cost‐effectiveness ratio (ICER)). Relevant data were extracted into a Microsoft Excel database.

A meta‐analysis of our data could not be conducted due to the heterogeneity in study designs, populations, time horizons, and outcomes of our included studies. Thus, only descriptive analyses were performed.

## 3 RESULTS

### 3.1 Study selection

**FIGURE 1:**
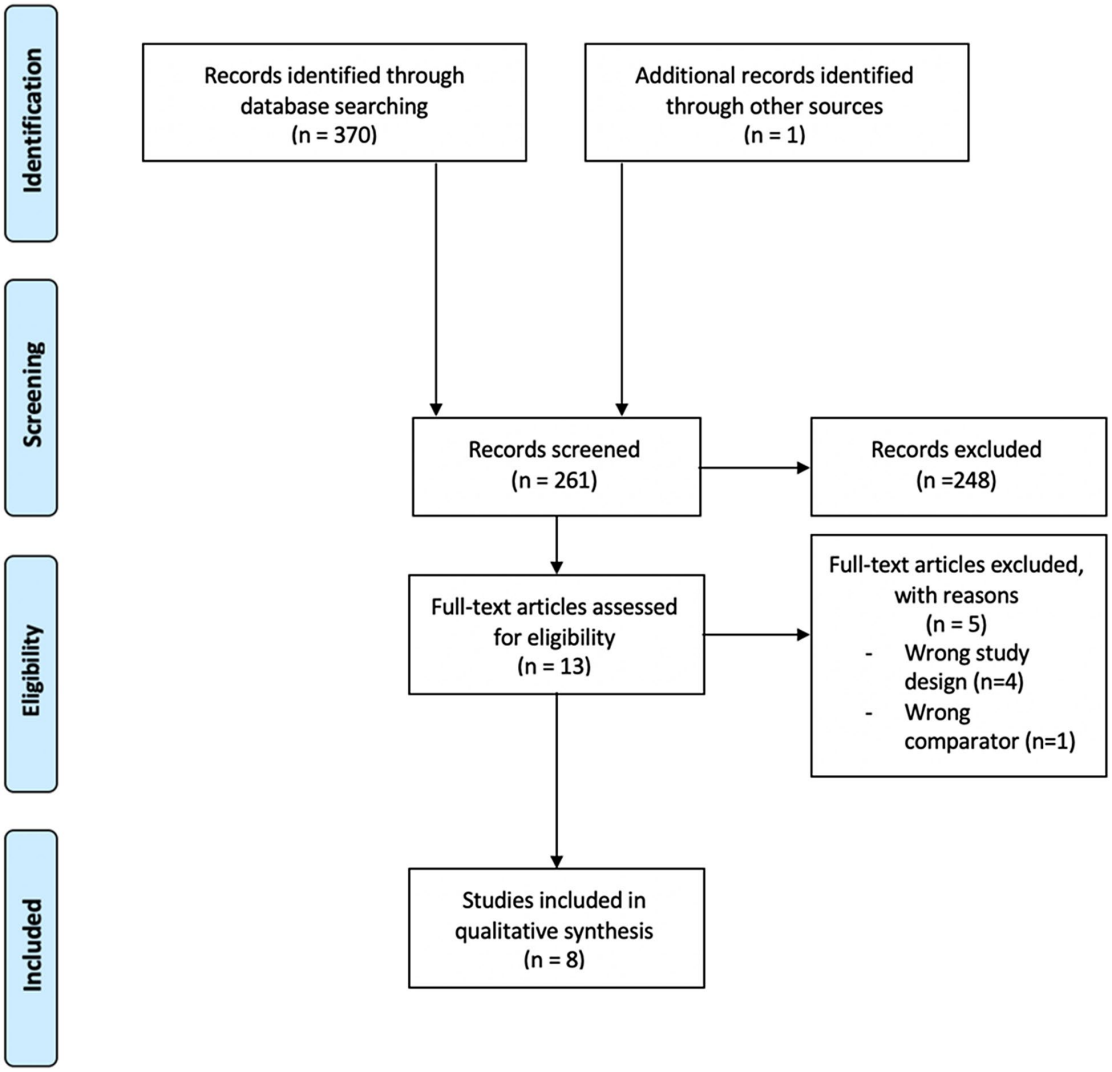
PRISMA flow diagram of study inclusion and selection. n = number of studies

Our search identified 371 citations. After the removal of 110 duplicates, we screened 261 titles/abstracts for eligibility. After title and abstract screening, 13 articles remained for full‐text screening. Of these 13 articles, eight studies fulfilled the study criteria and were included for further analysis. The main reasons for exclusion at the full‐text screening stage included wrong study design (n = 4) and wrong comparator used in study (n = 1). Figure 1 describes the process of study inclusion.

### 3.2 Study characteristics

Table 1 presents the characteristics of the eight included studies. The eight included studies were published between 2013 and 2018 and were conducted in Canada (n = 2),^4^, ^18^ the United Kingdom (n = 2),^24^, ^25^ Brazil (n = 1),^12^ Japan (n = 1),^26^ Italy (n = 1),^27^ and the United States (n = 1).^28^ All studies carried out cost‐utility analysis.^4^, ^12^, ^18^, ^24^, ^25^, ^26^, ^27^, ^28^ Three studies also examined cost per life‐year gained.^12^, ^26^, ^27^ One study also examined cost per UTI avoided per patient.^25^ Studies were conducted from the following perspectives: national health‐care system perspective (n = 5),^12^, ^24^, ^25^, ^27^, ^28^ public payer perspective (n = 2),^4^, ^18^ and payer perspective (n = 1).^26^ Six of the eight studies compared single‐use hydrophilic‐coated catheters to single‐use uncoated catheters.^12^, ^18^, ^25^, ^26^, ^27^, ^28^ Bermingham et al compared single‐use hydrophilic‐coated catheters to single‐use gel reservoir catheters, sterile uncoated catheters, and clean uncoated catheters.^24^ The 2019 HQO report compared single‐use hydrophilic‐coated catheters to both single‐use uncoated catheters and repeated‐use uncoated catheters.^4^

**TABLE 1:** Characteristics of economic evaluation studies examining hydrophilic‐coated vs uncoated catheters for individuals with spinal cord injury included for analysis

Author, year	Study type	Location	Population	Perspective	Time horizon	Intervention/Comparator	Results
Bermingham et al., 2013^24^	Cost‐utility	United Kingdom	Individuals with neurogenic bladder condition due to spinal cord injury	UK National Health Services	Lifetime horizon	Single‐use hydrophilic‐coated, single‐use gel reservoir, sterile uncoated, and clean uncoated catheters	Clean uncoated catheters (1 per week) were cost‐effective at a £20 000 threshold
							Excluding clean uncoated catheters, single‐use gel reservoir catheters were cost‐effective compared to hydrophilic‐coated catheters (ICER: £3071 per QALY gained)
Clark et al*.,* 2015^25^	Cost‐effectiveness	United Kingdom	Individuals with spinal cord injury and chronic urinary retention	UK National Health Services	Lifetime horizon	Single‐use hydrophilic‐coated catheters and uncoated catheters	Hydrophilic‐coated catheters were cost‐effective with an ICER of £6100 per QALY gained, £3300 per life‐year gained, and £79 per UTI event avoided
Hakansson et al*.,* 2016^28^	Cost‐effectiveness	United States	Inpatients with spinal cord injury and chronic urinary retention	US health‐care system	Lifetime horizon	Single‐use hydrophilic‐coated and uncoated catheters	Single‐use hydrophilic‐coated catheters reduced long‐term health‐care costs
							Hydrophilic‐coated catheters were more cost‐effective than uncoated catheters, saving $10 184, gaining 0.55 QALY, and avoiding 16 UTIs per person on average
Rognoni and Tarricone, 2017^27^	Cost‐effectiveness	Italy	Individuals with spinal cord injury who use intermittent catheterization in a community setting	Italian health‐care service	Lifetime horizon	Single‐use hydrophilic‐coated and single‐use uncoated catheters	Single‐use hydrophilic‐coated catheters were cost‐effective compared to uncoated catheters (ICER: €24,405 per QALY gained and €20,761 euros per life‐year gained)
Watanabe et al., 2017^26^	Cost‐effectiveness	Japan	Inpatients with spinal cord injury and chronic urinary retention	Japanese payer	Lifetime horizon	Single‐use hydrophilic‐coated and uncoated catheters	Hydrophilic‐coated catheters were cost‐effective compared to uncoated catheters with an ICER of ¥3,826,351 per QALY gained and ¥1,639,562 per life‐year gained
Truzzi et al*.,* 2018^12^	Cost‐effectiveness	Brazil	Inpatients and outpatients with spinal cord injury	Brazilian public health‐care system	Lifetime horizon	Single‐use hydrophilic‐coated and uncoated catheters	Single‐use hydrophilic‐coated catheters were cost‐effective in comparison to uncoated catheters with an ICER of R$57,432 per life‐year gained and R$122,300 per QALY gained
							When accounting for only UTIs, hydrophilic‐coated catheters reduced UTI risk by 6% for an additional cost of R$31,240
Welk et al., 2018^18^	Cost‐effectiveness	Canada	Individuals with neurogenic bladder dysfunction due to spinal cord injury	Canadian public payer (Ontario Ministry of Health and Long‐term Care)	Lifetime horizon	Single‐use hydrophilic‐coated and uncoated catheters	Hydrophilic‐coated catheters were cost‐effective compared to uncoated catheters (ICER: $66 462 per QALY gained)
Health Quality Ontario, 2019^4^	Cost‐utility	Canada	Inpatients and outpatients with chronic urinary retention due to spinal cord injury	Canadian public payer (Ontario Ministry of Health and Long‐term Care)	5‐year	Single‐use hydrophilic‐coated, repeated‐use uncoated (1/day and 1/week), and single‐use uncoated catheters	ICER for single‐use hydrophilic‐coated vs repeated‐use uncoated (1/wk) catheters: $3.5 million per QALY gained
							ICER for single‐use hydrophilic‐coated vs single‐use uncoated catheters: $3 million per QALY gained

### 3.3 Quality assessment

Table 2 outlines the methodological quality assessment of the included economic evaluation studies based on the Drummond checklist. Overall, the eight included studies were well reported and no main methodological issues were identified. However, most of the included studies did not clearly state or justify the perspective of the analysis or justify the type of economic evaluation used in the study.

**TABLE 2:** Results of the economic evaluation quality assessment by Drummond et al^21^

	Welk (2018)^18^	Watanabe (2017)^26^	Truzzi (2018)^12^	Rognoni (2017)^27^	Clark (2016)^25^	Bermingham (2013)^24^	Hakkanson (2016)^28^	HQO (2019)^4^
*Study design*								
1. The research question is stated	Yes	Yes	Yes	Yes	Yes	Yes	Yes	Yes
2. The economic importance of research question is stated	Yes	Yes	No	Yes	Yes	Yes	Yes	Yes
3. The viewpoint(s) of the analysis is clearly stated and justified	Not clear	No	No	No	No	No	No	No
4. The rationale for choosing the alternative programmes or interventions compared is stated	Yes	No	Yes	Yes	Yes	Yes	Yes	Yes
5. The alternatives being compared are clearly described	Yes	No	Yes	Yes	Yes	Yes	Yes	Yes
6. The form of economic evaluation used is stated	Yes	Yes	Yes	Yes	Yes	Yes	No	Yes
7. The choice of form of economic evaluation is justified in relation to the questions addressed	No	No	No	No	No	No	No	Yes
*Data collection*								
8. The source(s) of effectiveness estimates used are stated	Yes	Yes	Yes	Yes	Yes	Yes	Yes	Yes
9. Details of the design and results of effectiveness study are given (if based on a single study)	Not appropriate	Not appropriate	Not appropriate	Not appropriate	Not appropriate	Not appropriate	Not appropriate	Not appropriate
10. Details of the method of synthesis or meta‐analysis of estimates are given (if based on an overview of a number of effectiveness studies)	Yes	Yes	Yes	Yes	Yes	Yes	Yes	Yes
11. The primary outcome measure(s) for the economic evaluation is clearly stated	Yes	No	Yes	Yes	Yes	Yes	Yes	Yes
12. Methods to value health states and other benefits are stated	Yes	Yes	Yes	Yes	Yes	Yes	Yes	Yes
13. Details of the subjects from whom valuations were obtained are given	Yes	No	No	No	No	No	No	Yes
14. Productivity changes (if included) are reported separately	Yes	Not appropriate	Not appropriate	Not appropriate	Not appropriate	Not appropriate	Not appropriate	Not appropriate
15. The relevance of productivity changes to the study question is discussed	Yes	Not appropriate	Not appropriate	Not appropriate	Not appropriate	Not appropriate	Not appropriate	Not appropriate
16. Quantities of resources are reported separately from their unit costs	Yes	Yes	Yes	Yes	Yes	Yes	Yes	Yes
17. Methods for the estimation of quantities and unit costs are described	Yes	Yes	Yes	Yes	Yes	Yes	Yes	Yes
18. Currency and price data are recorded	Yes	Yes	Yes	Yes	No	Yes	Yes	Yes
19. Details of currency of price adjustments for inflation or currency conversion are given	Yes	No	Not clear	No	No	No	Yes	Yes
20. Details of any model used are given	Yes	Yes	Yes	Yes	Yes	Yes	Yes	Yes
21. The choice of model used and the key parameters on which it is based are justified	Yes	No	No	No	Yes	Yes	Yes	Yes
*Analysis and interpretation of results*								
22. Time horizon of costs and benefits is stated	Yes	Yes	Yes	Yes	Yes	Yes	Yes	Yes
23. The discount rate(s) is stated	Yes	Yes	Yes	Yes	Yes	Yes	Yes	Yes
24. The choice or rate(s) is justified	Yes	No	No	Yes	Yes	No	Yes	Yes
25. An explanation is given if costs or benefits are not discounted	Not appropriate	Not appropriate	Not appropriate	Not appropriate	Not appropriate	Not appropriate	Not appropriate	Not appropriate
26. Details of statistical tests and confidence intervals are given for stochastic data	No	No	No	No	Yes	No	No	Yes
27. The approach to sensitivity analysis is given	Yes	Yes	Yes	Yes	Yes	Yes	Yes	Yes
28. The choice of variables for sensitivity analysis is justified	No	Yes	No	Yes	Yes	Yes	Yes	Yes
29. The ranges over which the variables are varied are stated	Yes	Yes	Yes	Yes	Yes	Yes	Yes	Yes
30. Relevant alternatives are compared	Yes	Yes	Yes	Yes	Yes	Yes	Yes	Yes
31. Incremental analysis is reported	Yes	Yes	Yes	Yes	Yes	Yes	Yes	Yes
32. Major outcomes are presented in a dissaggregated as well as aggregated form	Yes	Yes	Yes	Yes	Yes	Yes	Yes	Yes
33. The answer to the study question is given	Yes	Yes	Yes	Yes	Yes	Yes	Yes	Yes
34. Conclusions follow from the data are reported	Yes	Yes	Yes	Yes	Yes	Yes	Yes	Yes
35. Conclusions are accompanied by the appropriate caveats	Yes	Yes	Yes	Yes	Yes	Yes	Yes	Yes
**Stated source(s) of funding**	Yes	Yes	Yes	Yes	Yes	Yes	No	No
**Stated conflict(s) of interest**	Yes	Yes	Yes	Yes	Yes	Yes	No	No

### 3.4 Summary of the study methods and results

The results of the included studies are summarized in Table 1.

Several economic evaluations have been conducted regarding the use of different types of catheters for individuals with spinal cord injury. The first economic evaluation of single‐use and repeated‐use intermittent catheters was conducted by Bermingham et al for the National Institute for Health and Clinical Excellence (NICE) in the United Kingdom.^24^ This cost‐utility analysis used a Markov model to examine costs, quality‐adjusted life years (QALYs), and incremental cost per QALY gained over a lifetime horizon from a UK National Health Services perspective. The Markov model examined the short‐term sequelae of UTI and assumed the same risk of UTI between different catheter types. The input for the Markov model was based largely on the authors’ systematic review and meta‐analysis of randomized control trial and random crossover trial papers published between 2002 and 2011. Bermingham et al found that hydrophilic‐coated catheters were not cost‐effective when compared to uncoated catheters reused for the entire week (one per week) or daily (one per day).^24^ However, excluding uncoated catheter reuse, hydrophilic‐coated catheters was observed to be less costly and have better QALYs. Sensitivity analyses conducted by the authors demonstrated that clean uncoated catheters would not be the most cost‐effective bladder management option if the patient used more than one clean uncoated catheter per day.

The Bermingham et al^24^ paper was quickly followed by a cost‐effectiveness study conducted by Clark et al,^25^ also based in the United Kingdom. The study was conducted over a lifetime horizon from a UK National Health Services perspective. Clark et al used the model by Bermingham and colleagues as a foundation, but examined long‐term sequelae of UTI and applied a UTI risk reduction rate of 21% for individuals who used hydrophilic‐coated catheters in comparison to those who used uncoated catheters. Additionally, Clark et al used randomized control trials of solely adult spinal cord injury participants as their parameter input. The authors examined costs, QALYs, cost per life‐year gained, and cost per UTI event. At a cost‐effectiveness threshold of £20 000‐30 000 per QALY gained, the authors found hydrophilic‐coated catheters to be cost‐effective. Subsequent economic evaluation studies on this topic used Markov models based on the model developed by Bermingham et al,^4^, ^27^, ^28^ the model developed by Clark et al,^12^, ^26^ or both.^18^

Another cost‐effectiveness analysis was conducted by Hakansson et al^28^ The authors used a Markov model, based on the model developed by Bermingham et al, that included various health states for potential complications including epididymitis, strictures, and bladder stones. The study did not distinguish between costs of the different catheter types, except that uncoated catheters had an additional cost of $0.13 per catheter for the cost of lubricant. Hakansson et al found that single‐use hydrophilic‐coated catheters reduced long‐term health‐care costs and were more cost‐effective than uncoated catheters. Their sensitivity analyses revealed that single‐use hydrophilic‐coated catheters should remain cost‐effective until the cost of a single hydrophilic‐coated catheter becomes equal to or greater than $2.84.

In 2017, Rognoni and Tarricone also conducted a cost‐effectiveness analysis comparing single‐use hydrophilic‐coated and single‐use uncoated catheters.^27^ They use a Markov model, based on the one developed by Bermingham et al, to investigate the costs, life‐years gained, and QALYs gained for the two types of catheters from the Italian health‐care service perspective over a lifetime horizon. This study examined the short‐term consequences of UTIs and hematuria. They also included model parameters for other infections and inflammations relevant to catheterization including epididymitis/orchitis, urethritis, prostatitis, strictures, false passage, and bladder stones. The source of data for model input consisted of health‐care resource utilization derived from an e‐survey completed by a group of 25 urologists and neuro‐urologists. At Italian cost‐effectiveness threshold values of €25 000‐40 000, €36 500, €60 000, and €66 400 per QALY gained and at the United Kingdom‐specific threshold value recommended by NICE of £20 000‐30 000 per QALY gained, Rognoni and Tarricone found single‐use hydrophilic‐coated catheters to be cost‐effective compared to uncoated catheters.

A fourth economic evaluation study was conducted by Watanabe et al and sought to investigate the cost‐effectiveness of single‐use hydrophilic‐coated catheters vs uncoated catheters.^26^ The Markov model was based on the model developed by Clark et al, but with Japanese data derived from clinician surveys, published literature, and national statistics. The model examined cost per QALY gained, cost per life‐year gained, and cost per UTI event avoided over a lifetime horizon from a Japanese payer perspective. Applying a cost‐effectiveness threshold of ¥6 700 000 per QALY gained (US$55 372/QALY), Watanabe et al found hydrophilic‐coated catheters to be cost‐effective compared to uncoated catheters.

In 2018, Truzzi et al conducted a cost‐effectiveness analysis from a Brazilian public payer perspective over a lifetime horizon to examine the cost per QALY gained of single‐use hydrophilic‐coated catheters vs uncoated catheters.^12^ Their Markov model was based on the model used by Clark et al, but included separate health states for first‐ and second‐line antibiotic‐resistant UTIs. The authors conducted two scenario analyses, one including all possible adverse events (ie, UTIs, bladder stones, kidney stones, urethral injury, and urosepsis) and one including only UTIs. Accounting for all possible adverse events and applying a cost‐effectiveness threshold of R$147 000 per QALY gained, the authors found single‐use hydrophilic‐coated catheters to be cost‐effective in comparison to uncoated catheters. When accounting for only UTIs, hydrophilic‐coated catheters reduced UTI risk by 6% for an additional cost of R$31 240.

A cost‐effective analysis was conducted by Welk et al in 2018.^18^ The study used a Markov model, derived from the one used by Clark et al, to examine the incremental costs, QALYs, life‐year gained, and number of UTIs avoided over a lifetime horizon from a Canadian public payer perspective (ie, perspective of the Ontario Ministry of Health and Long‐term Care). This Markov model was based on the model developed by Clark et al to investigate the long‐term sequelae of UTI and used data from previous meta‐analyses and provincial and national data from the government of Ontario and Statistics Canada. Welk et al used cost‐effectiveness thresholds of $20 000‐100 000 per QALY gained and $50 000‐100 000 per QALY gained and found hydrophilic‐coated catheters to be cost‐effective compared to uncoated catheters.

Finally, an economic evaluation studying various catheter types was conducted by HQO in 2019.^4^ The study included data for inpatients and outpatients with chronic urinary retention due to spinal cord injury and examined the incremental cost per QALY of hydrophilic‐coated vs uncoated catheters over a 5‐year time horizon from the perspective of the Ontario Ministry of Health and Long‐term Care. The HQO report adapted the Markov model used by Bermingham et al, assuming equal risk of complications between the different catheter types and examining short‐term consequences of complications such as UTIs and hematuria. At a cost‐effectiveness threshold of $100 000 per QALY gained, the HQO report found single‐use uncoated catheters to be 100% cost‐effective, but not hydrophilic‐coated catheters.

## 4 DISCUSSION

Our study sought to assess the cost‐effectiveness of hydrophilic‐coated catheters compared to other types of urinary catheters among the spinal cord injury population. We identified eight studies that reported the cost‐effectiveness of hydrophilic‐coated catheters in the spinal cord injury population, evaluating seven different types of catheters.

Using ICER per QALY gained as the primary measure of cost‐effectiveness, the evidence surrounding the cost‐effectiveness of hydrophilic‐coated catheters in comparison to other types of catheters varied. Five studies observed hydrophilic‐coated catheters to be cost‐effective compared to uncoated catheters.^12^, ^18^, ^25^, ^26^, ^27^ Two studies found hydrophilic‐coated catheters to be not cost‐effective compared to uncoated catheters^4^, ^24^ and one study estimated that hydrophilic‐coated catheters reduced the long‐term health‐care costs compared to uncoated catheters.^28^

Several study‐related factors help to explain the discrepancy in results observed. First, the results appear to depend on the primary treatment comparator in the economic evaluations. In the study by Bermingham et al, uncoated catheters reused for a full week was selected as the comparator to all other treatment options.^24^ None of the other treatment comparators evaluated were considered cost‐effective when compared to repeated‐use uncoated catheters. This is not surprising given the considerable cost difference between a single uncoated catheter, vs the 25‐35 single‐use hydrophilic‐coated catheters that patients would require in a week. However, when repeated‐use uncoated catheters were excluded from the analysis, hydrophilic‐coated catheters were considered less costly with better outcomes compared to single‐use uncoated catheters and gel reservoir was considered cost‐effective compared to hydrophilic‐coated catheters.^24^ Similarly, in the HQO report, the reference case analysis compared hydrophilic‐coated catheters to uncoated catheters reused for a full week.^4^ The ICER decreased from $3.7 million per QALY to $3.1 million per QALY when repeated‐use uncoated catheters were excluded. All other studies identified in our review used single‐use uncoated catheters as the treatment comparator. As noted in several of the studies included in our review, including the study by Bermingham et al, the reuse of uncoated catheters is not supported by health‐care governing organizations and is considered off‐label use.^18^, ^24^, ^27^ Many countries have adapted single‐use uncoated catheters as the minimum standard for clean intermittent catheterization. Thus, a comparison of approved intermittent catheters would result in all studies observing hydrophilic‐coated catheters to be cost‐effective compared to uncoated catheters with the exception of the HQO report.

Second, the discrepancy in the cost‐effectiveness outcomes of our included studies may be related to whether long‐term health impacts were considered. The model design for the economic evaluations identified in our systematic review can be separated into two categories: studies that evaluated short‐term health effects^4^, ^24^, ^27^ and studies that considered the long‐term health implications of using different catheter types.^12^, ^18^, ^25^, ^26^, ^28^ Studies focused on the long‐term implications considered the impact of secondary complications on renal function, which increased the benefits associated with reducing UTIs and other renal complications. As a result, studies including the long‐term impact on renal function typically had greater incremental QALYs (between 0.26 and 0.72)^12^, ^18^ in comparison to studies examining short‐term impact (0.077‐0.18).^4^, ^24^ This difference could partially explain the discrepancy in cost‐effectiveness outcomes, with larger incremental QALYs resulting in lower incremental cost per QALY. The only exception was the study by Rogoni et al an economic evaluation of short‐term outcomes that observed an incremental QALY of 0.84.^27^ This exception can be explained by the measure of reduction in symptomatic UTI between hydrophilic‐coated catheters and uncoated catheters used in the model inputs. For the Bermingham et al paper and the HQO report, the measure of benefit was the difference in number of individuals experiencing at least one UTI.^4^, ^24^ Moreover, Rogoni et al incorporated the difference in the average number of UTIs experienced per person per month.^27^ The average number of UTIs was selected by Rogoni et al to provide a more comprehensive analysis of the benefits of hydrophilic‐coated catheters resulting in greater accuracy in cost and quality of life results.^27^

Finally, the unit cost of treatment may have notably impacted the incremental cost of hydrophilic‐coated catheters resulting in a discrepancy in the economic evaluation outcomes. Both Bermingham et al and Hakansson et al assumed similar unit costs for hydrophilic‐coated and uncoated catheters.^24^, ^28^ With the additional cost of lubricant for the administration of uncoated catheters, the total cost for treatment with uncoated was greater than hydrophilic‐coated catheters. As such, both studies observed a decrease in cost with hydrophilic‐coated catheters in comparison to uncoated catheters. The daily cost premium for hydrophilic‐coated catheters estimated in other economic evaluations ranged between $2.49 US dollars (£1.75 per day), reported by Clark et al,^25^ and $8.96 ($10.80 Canadian per day), reported by Welk et al^18^ The largest daily cost differential for catheters was reported by the HQO report at $24.24 per day ($29.20 Canadian per day.^4^ This cost differential resulted in a higher total lifetime incremental cost of $183,243 for hydrophilic‐coated catheter treatment compared to uncoated catheters.^4^ This is about 3.8 times higher than the cost differential reported by Welk et al examined in the same jurisdiction at the same time.^18^ In a single‐payer health‐care system such as Canada, it seems likely that the per unit costs could be significantly reduced with large‐scale purchase agreements.

Furthermore, we found that there appeared to be evidence for additional economic benefits when a societal perspective was considered. For example, Welk et al found that hydrophilic‐coated catheters dominated uncoated catheters in terms of short‐ and long‐term sick leaves, early retirement, and early death, implying that societal costs for users of hydrophilic‐coated catheters are less than those for users of uncoated catheters.^18^ These additional benefits, including savings in societal costs, would be lost if decision makers only look at the contrasting results.

In terms of future directions, further studies are needed to evaluate the short‐term and long‐term effects of hydrophilic‐coated catheter use on UTI. A systematic review and meta‐analysis of intermittent catheterization with hydrophilic‐coated and non‐hydrophilic‐coated catheters noted that many studies were randomized control trials with small samples and had attrition bias as a result of greater dropouts in the hydrophilic‐coated catheter arm.^27^ Another systematic review and meta‐analysis on the same topic, noted similar limitations as well as insufficient study sample sizes and no studies with observation periods beyond 1 year.^4^ Thus, there is a need for better evidence on this important clinical outcome. In addition to studies on hydrophilic‐coated catheter use and UTI risk, there is also a need for additional evidence on the impact of hydrophilic‐coated catheter use on long‐term renal function. The first economic evaluation model incorporating long‐term progression in renal function was developed with the guidance of urologists and spinal cord injury rehabilitation specialists.^25^ Since this study, the same model has been adapted in three other countries by other research teams^12^, ^18^, ^26^ suggesting that this model may be a better reflection of clinical reality. However, the recent HQO report excluded long‐term renal function outcomes following consultation with expert opinion and a NICE report on childhood UTI.^4^ Studies on the chronic use of hydrophilic‐coated catheters on renal function would be beneficial in providing evidence on the long‐term impacts of the use of different catheter types and provide stronger justification on whether to limit an economic evaluation to short‐term outcomes. There is also a need for greater transparency on the acquisition cost of hydrophilic‐coated catheters. As described earlier, the large range in unit costs used in different economic evaluations may be causing a large discrepancy in incremental cost and resulting in vastly different incremental cost per QALYs. Given that individuals are estimated to use between three to five catheters per day, even small differences in unit cost of the device will have large incremental costs over a lifetime. Finally, the inclusion of repeated‐use uncoated catheters as a comparator requires a larger discussion and guidance by government health‐care agencies on the appropriateness of off‐label interventions in economic evaluations. This will likely require a broader discussion on the reasons why individuals are administering medical interventions beyond its intended use and may broach the subject of health‐care accessibilities, barriers, and funding.

A strength of our study was the use of the Preferred Reporting Items for Systematic Reviews and Meta‐Analyses (PRISMA) guidelines and the Drummond checklist for economic evaluations to identify, assess, and critically examine our included studies. Nevertheless, our study does have several limitations that should be considered. First, we did not search gray literature databases, which may have led to the exclusion of a few potentially relevant citations. Thus, it is possible that our search missed economic analyses conducted by government agencies or health‐care organizations. Second, whether hydrophilic‐coated catheters were considered cost‐effective for each study was dependent on the study author's interpretation of the results. For the most part, this interpretation was based on the common cost‐effectiveness thresholds reported in the country where the study was conducted. Third, most of our included studies have limited generalizability. The generalizability of the economic evaluation studies we included tended to be limited to the specific population specified in their respective study designs.

Our critical evaluation of the literature suggests that the evidence is pointing toward hydrophilic‐coated catheters being cost‐effective. The comparator intervention, inclusion of long‐term outcomes, and the unit cost of the catheter appear to influence the estimated cost‐effectiveness hydrophilic‐coated catheters. Our findings are relevant, particularly among the spinal cord injury population, who are often injured when they are young, and are expected to use intermittent catheters for decades.^1^ Financial support of hydrophilic‐coated catheters may improve the quality of life and reduce urinary infections, and thus reduce the significant number of patients that switch to indwelling catheters, a medically inferior bladder management method.^29^

## CONFLICT OF INTEREST

Ms Xi reports an unrestricted grant by Spinal Cord Injury Ontario and is a part of the Spinal Cord Injury Ontario Pee For Free working group steering committee. Ms Pakosh reports an unrestricted grant by Spinal Cord Injury Ontario. Dr Elterman has nothing to disclose. Dr Welk has nothing to disclose. Dr Chan reports an unrestricted grant by Spinal Cord Injury Ontario and is a part of the Spinal Cord Injury Ontario Pee For Free working group steering committee.

## Funding information

This study was supported by Spinal Cord Injury Ontario through an unrestricted grant. The authors had independent control over study methods, analysis, and interpretation of the results. Spinal Cord Injury Ontario was provided with an earlier draft of the manuscript for review. Spinal Cord Injury Ontario did not have editorial control over the manuscript content

## APPENDIX A

### SEARCH STRATEGY

**Search Strategy: Ovid MEDLINE(R) ALL <1946 to October 18, 2019>**

1. [Question: What are the economics of intermittent catheter use for individuals with Spinal Cord Injury?]
2. [Population: Spinal Cord Injury/Neurogenic Bladder]
3. exp Spinal Cord Injuries/
4. exp Spinal Injuries/
5. exp Spine/ and trauma*.tw,kw.
6. exp Paraplegia/
7. exp Quadriplegia/
8. Urinary Bladder, Neurogenic/
9. ((spine or spinal or vertebra*) adj3 (injur* or contusion* or trauma* or transection* or laceration* or compression*)).tw,kw. (
10. (paraplegi* or quadriplegi* or quadripares* or tetraplegi*).tw,kw. (24,431)
11. (myelopath* adj2 (traumatic or post‐traumatic or post traumatic or compressive)).tw,kw.
12. (bladder* adj3 (neurogen* or neuropath*)).tw,kw.
13. or/3‐12
14. [Intervention: Catheters/Catheterization]
15. exp Catheters/
16. exp Catheterization/
17. (catheter* or self‐catheter*).tw,kw.
18. or/15‐17
19. [Outcomes: Economic/Cost Analysis]
20. economics/
21. "costs and cost analysis"/
22. cost‐benefit analysis/
23. "cost savings"/
24. "cost of illness"/
25. exp health care costs/
26. exp health expenditures/
27. exp economics, hospital/
28. exp economics, medical/
29. health care sector/
30. quality‐adjusted life years/
31. (cost* adj3 (analys* or compar* or measur* or effect* or benefi* or utilit* or saving* or variation* or minimization* or minimisation* or assess* or "out‐of‐pocket" or increment* or health care or healthcare or variance* or overall or averag* or direct or indirect or illness* or total* or data* or ratio* or outpatient* or inpatient* or patient* or clinic* or device* or "per life" or lifetime* or estimat* or differ* or burden*)).tw,kw.
32. ((econom* or financ* or budget*) adj3 (evaluat* or impact* or analys* or burden or consequence* or strateg* or model* or comparator*)).tw,kw.
33. (life year* adj3 (quality‐adjusted or quality adjusted)).tw,kw.
34. or/20‐33
35. 13 and 18 and 34

***************************
